# Retention and tissue-specific expression of uncoupling protein homoeologs in *Xenopus laevis*

**DOI:** 10.1242/bio.062691

**Published:** 2026-07-08

**Authors:** Erik Rollwitz, Martin Jastroch

**Affiliations:** Department of Molecular Biosciences, The Wenner-Gren Institute (MBW), Stockholm University, SE-106 91 Stockholm, Sweden

**Keywords:** Uncoupling proteins, Homoeologs, Amphibians

## Abstract

Mitochondrial uncoupling proteins (UCPs) play central roles in vertebrate energy metabolism, with UCP1 specializing as a thermogenic effector in placental mammals. Comparative genomics demonstrated that *UCP1*, *UCP2*, and *UCP3* originated prior to the emergence of endothermy, suggesting that their ancestral functions evolved in ectothermic vertebrates. Here, we investigated the genomic organization, conserved synteny, and mRNA expression of all three *UCP* paralogs and homoeologs in the allotetraploid amphibian *Xenopus laevis*. Comparative genome analyses revealed that all three *UCP* paralogs and their long (L) and short (S) homoeologs were retained following polyploidization, although local rearrangements were evident for neighboring genes at the *UCP2*/*UCP3* locus. Quantitative expression analyses across adult frog tissues revealed pronounced tissue specificity, with predominant expression of *UCP1* and *UCP2* in kidney, *UCP3* in muscle, and differences in *UCP2* homoeolog expression levels. Together, these findings establish a framework for UCPs in an amphibian model organism, primarily linking them to energetically demanding tissues.

## Teaser

**Summary:** Complete retention and tissue-specific expression of uncoupling protein homoeologs in *Xenopus laevis* provide an updated framework for UCP organization in ectothermic vertebrates.

## INTRODUCTION

Uncoupling proteins (UCPs) are members of the mitochondrial solute carrier family 25 (SLC25) and reside in the mitochondrial inner membrane, where they impact energy transduction and metabolic fluxes (Walker and Runswick, 1993). In mammals, UCP1 is best known for its role in adaptive non-shivering thermogenesis in brown and beige adipose tissue, where it uncouples the proton motive force from ATP synthesis to dissipate chemical energy as heat and to accelerate oxidation processes (Gaudry et al., 2019). In contrast, the physiological roles of the related paralogs UCP2 and UCP3 are less well defined and are thought to encompass functions in mitochondrial substrate handling, redox control, immune regulation, and metabolic plasticity (Gaudry and Jastroch, 2019).

Comparative genomic analyses have demonstrated that UCP1, UCP2, and UCP3 arose early in vertebrate evolution, as they are also found in teleost fishes (Jastroch et al., 2005). Thus, the three paralogs must have been present before the divergence of ray- and lobe-finned fishes. This timing precedes the emergence of endothermy and the onset of thermogenic functionality of UCP1 (Keipert et al., 2024), indicating that ancestral UCPs must have fulfilled functions unrelated to heat production. Nevertheless, most functional insights into UCP biology derive from studies in endothermic mammals, leaving the physiological roles of UCPs in ectothermic vertebrates largely unresolved. Investigating UCPs in fish and other ectotherms therefore offers a unique opportunity to disentangle ancestral mitochondrial functions from mammal-specific specializations. While fish are fully aquatic, amphibians represent the next evolutionary stage by temporarily leaving the aquatic environment during adulthood. Amphibians are exposed to pronounced environmental fluctuations throughout their life cycle, including changes in temperature, hydration, and nutrient availability. Organs that play central roles in osmoregulation and metabolic homeostasis, such as the kidney and skeletal muscle, are therefore subject to substantial energetic demands. Previous work identified *UCP* genes in the genome of the diploid *Xenopus tropicalis* and demonstrated conserved synteny of *UCP1* with its flanking genes relative to mammals and teleost fishes (Trzcionka et al., 2008). Limitations in genome assemblies at the time precluded a clear distinction between the adjacent *UCP2* and *UCP3* genes, creating an artificial sequence mix dubbed ‘UCP2/3’. Early expression analyses detected ‘UCP2/3’ transcripts in the African clawed frog (*Xenopus laevis*) tissues using hybridization techniques (northern blotting), showing a rather ubiquitous tissue expression pattern. Probing the tissues with a *X. laevis UCP1* fragment, however, failed to detect any transcripts.

Physiological studies in the cane toad (*Bufo marinus*) reported cold- and fasting-induced alterations of ‘UCP2/3’ expression in amphibian tissues, suggesting a potential role for UCPs in adaptive metabolic responses in ectotherms (Trzcionka et al., 2008). However, it remains unclear whether such responses primarily reflect conserved functions of UCPs as mitochondrial carrier proteins or represent lineage-specific adaptations in amphibians.

**Fig. 1.**
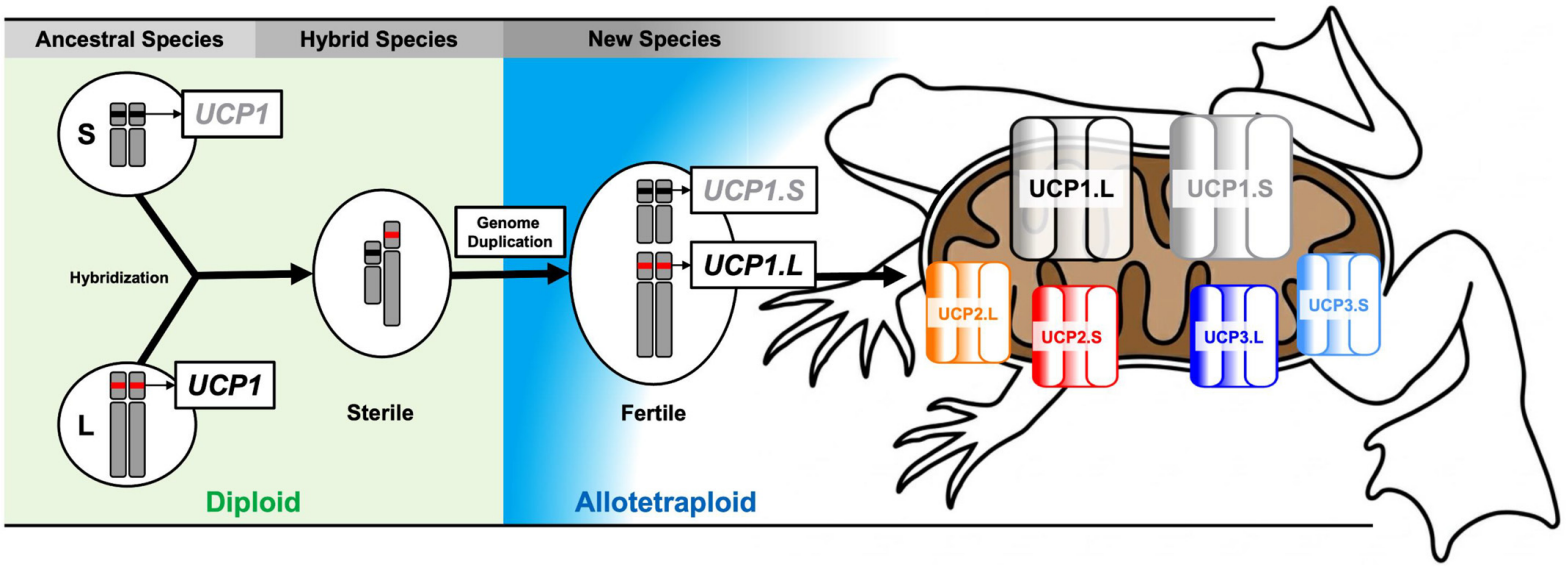
**Polyploid origin and homoeology in *X. laevis*.** Scheme depicting allotetraploid genome evolution in *X. laevis* and relation to mitochondrial uncoupling proteins. Hybridization between two extinct diploid progenitors followed by whole-genome duplication gave rise to L and S subgenomes, resulting in homoeologous gene pairs such as *UCP1.L* and *UCP1.S*. This evolutionary history underlies the presence of paralogous and homoeologous copies of uncoupling protein genes analyzed in this study. The frog outline picture was generated with Perplexity AI.

With the availability of improved *X. laevis* genome assemblies and annotations, it is now possible to reassess *UCP* gene organization, retention, and expression at homoeolog-specific resolutions. *X. laevis* represents a particularly informative system for such evolutionary and functional analyses. Members of the genus *Xenopus* exhibit remarkable variation in genome ploidy, ranging from diploid to dodecaploid species (Abu-Daya et al., 2012). *X. laevis* is allotetraploid, which arose through haploid gamete hybridization between two extinct diploid progenitor species, followed by whole-genome duplication (Fig. 1; Session et al., 2016; Tinsley et al., 1996). As a consequence, most protein-coding genes are present as pairs of homoeologs residing on the L (long) and S (short) subgenomes. Although the two subgenomes have remained largely distinct since polyploidization, they have evolved asymmetrically, with the S subgenome exhibiting higher rates of gene loss, rearrangement, and altered gene expression (Session et al., 2016). Functional studies in *X. laevis* must therefore account for differential retention and expression of L and S homoeologs, as these patterns can provide key insights into gene dosage constraints, subfunctionalization, and evolutionary importance.

In the present study, we combined comparative genomics and gene expression analyses to characterize UCPs in *X. laevis*. We first examined the conserved synteny and genomic organization of *UCP1*, *UCP2*, and *UCP3* and their L and S homoeologs relative to human and zebrafish orthologs. We then quantified homoeolog-specific *UCP* expression across adult frog tissues. Together, this integrative approach establishes a genomic and physiological framework for understanding UCP biology in an allotetraploid, ectothermic vertebrate and provides a foundation for future mechanistic and evolutionary studies.

## RESULTS AND DISCUSSION

### A comparative framework for *UCP1*, *UCP2*, and *UCP3* homoeologs in *X. laevis*

Understanding the evolutionary history and physiological roles of UCPs requires integrating genomic organization with tissue-specific expression patterns in phylogenetically informative model organisms. In this study, we provide a comprehensive analysis of *UCP1*, *UCP2*, and *UCP3* genomic organization and gene expression in the allotetraploid amphibian *X. laevis*, resolving paralogous and homoeologous gene retention, conserved synteny, and tissue expression in adult frogs. We establish a framework that highlights conserved and divergent aspects of UCP biology independent of mammalian specialization.

### Conserved synteny and homoeolog retention of *UCP* loci in *X. laevis*

**Fig. 2.**
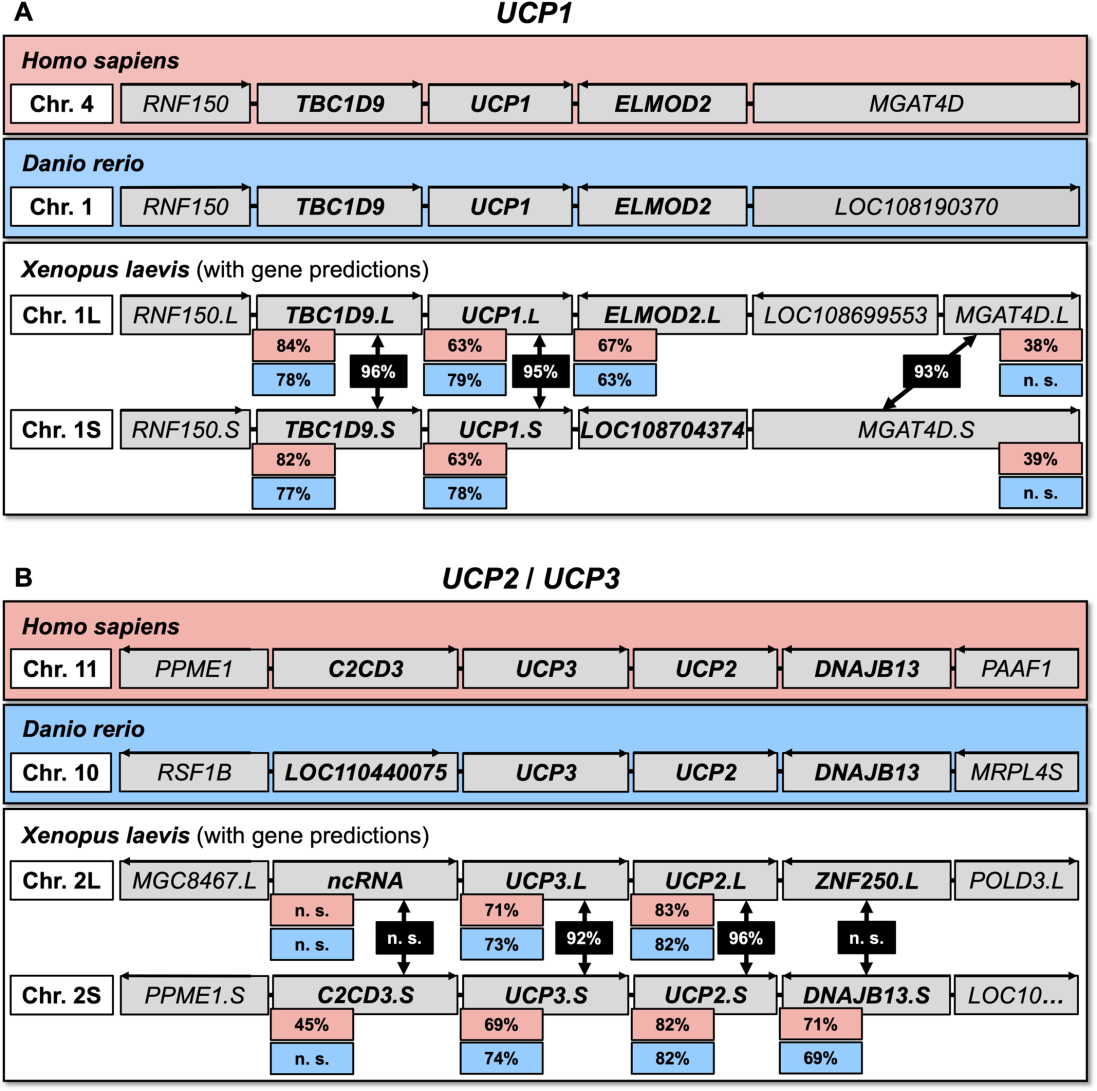
**Conserved synteny of *UCP* loci across vertebrates.** Conserved synteny of (A) *UCP1* and (B) *UCP2*/*UCP3* genomic regions in human (*Homo sapiens*), zebrafish (*Danio rerio*), and African clawed frog (*X. laevis*). Gene order, transcriptional orientation, and amino acid identity values highlight strong conservation of the *UCP1* locus across vertebrates, while the *UCP2*/*UCP3* region shows greater local rearrangement between species and between L and S subgenomes in *X. laevis*. Gene names (boxed in gray) with genomic orientation (arrows above gene names). Amino acid identity between *X. laevis* L and S homoeologs (boxed in black) and human (red) and zebrafish (blue) orthologs. ncRNA, noncoding RNA; n.s., no substantial similarities found.

Comparative genomic analyses revealed that all three major *UCP* paralogs (*UCP1*, *UCP2*, and *UCP3*) are retained in the *X. laevis* genome as pairs of L and S homoeologs. The *UCP1* locus exhibited high conservation of syntenic organization relative to human and zebrafish orthologs, including preservation of flanking genes (Fig. 2A). Both *UCP1.L* and *UCP1.S* displayed high amino acid identity and minimal divergence from each other (about 5%), as well as to zebrafish *UCP1* (about 21%) and human *UCP1* (about 37%). This conservation suggests that *UCP1* is subject to particularly strong evolutionary constraints, likely reflecting essential metabolic functions predating the emergence of endothermy.

In contrast, the *UCP2*/*UCP3* locus showed greater local genomic rearrangement (Fig. 2B). Several neighboring genes present in mammalian and teleost loci were absent or repositioned in one homoeologous chromosome but retained in the other. Such asymmetry is consistent with the known differential evolution of the L and S subgenomes in *X. laevis* (Session et al., 2016). Importantly, however, no loss of *UCP* coding sequences was observed, underscoring selective retention following allotetraploidization, despite broader genome restructuring. The high amino acid similarity between homoeologs further suggests that post-duplication divergence has occurred primarily at the regulatory level rather than the coding level.

### Tissue-specific mRNA expression of *UCP* paralogs and homoeologs in adult frogs

**Fig. 3.**
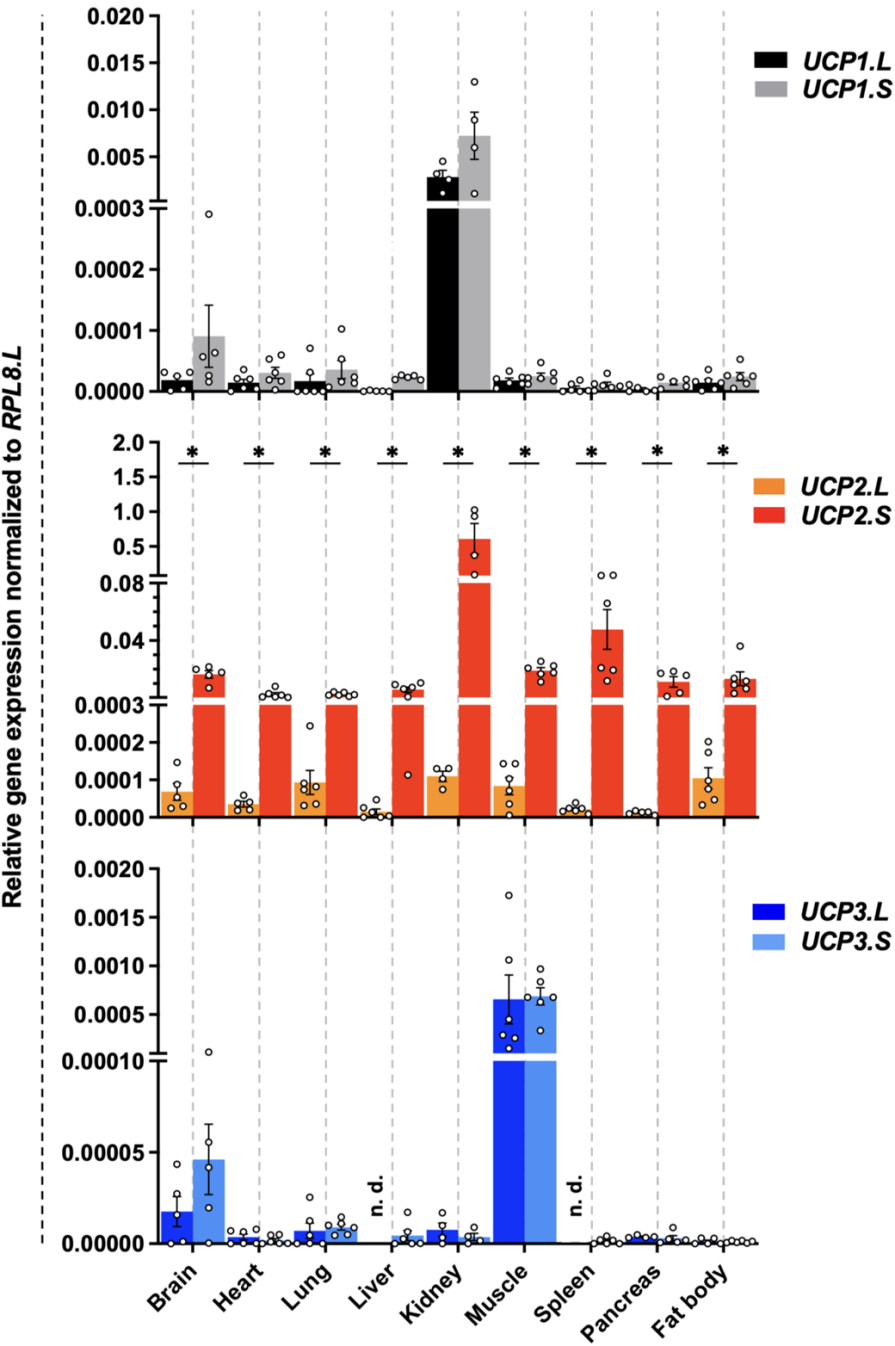
**Tissue-specific mRNA expression of *UCP* paralogs and homoeologs in adult *X. laevis*.** Relative mRNA expression of *UCP1*, *UCP2*, and *UCP3.L* and *S* homoeologs in adult *X. laevis* tissues measured by quantitative PCR. Tissue averages are shown as mean±s.e.m. in the bar charts and error bars. Y-axis breaks were introduced to depict differences of low expression levels. White round symbols represent individual samples (*n*) measured in duplicate. Mann–Whitney *U* tests were performed and corrected for multiple comparisons using the Holm–Sidak method; *n*=4–6. *, significant difference between homoeologs within the same tissue; n.d., not detectable.

Quantitative expression analyses revealed distinct tissue-specific expression patterns for *UCP* paralogs in adult *X. laevis*. Both *UCP1* homoeologs were predominantly expressed in the kidney, with barely detectable expression in other metabolically active tissues (Fig. 3). This pattern contrasts sharply with the adipose-restricted expression of UCP1 in mammals and supports the view that the canonical thermogenic role of UCP1 represents a derived feature of placental mammals (Keipert et al., 2024). Notably, *UCP1* in fish is also highly expressed in kidney tissue, although the highest levels were found in the liver (Jastroch et al., 2005).

Similarly, *UCP2* expression was also highest in the kidney. In particular, the S homoeolog is significantly more highly expressed across tissues. In mammals, *UCP2* has been implicated in regulating substrate utilization and reactive oxygen species production (Brand and Esteves, 2005; Vozza et al., 2014), functions that are highly relevant to renal physiology. The kidney is among the most energy-intensive organs in vertebrates due to continuous ion transport and osmotic regulation (O'Connor, 2006; Wang et al., 2010), and the enrichment of *UCP1* and *UCP2* in this tissue suggests conserved roles in supporting renal metabolic homeostasis.

In contrast, *UCP3* homoeologs were most highly expressed in skeletal muscle, with minimal expression in other tissues. This muscle-specific pattern mirrors that observed in other vertebrate lineages, such as fish (Jastroch et al., 2005), birds (Rey et al., 2010), marsupials (Jastroch et al., 2004) and eutherians (Boss et al., 1997), indicating conservation of UCP3 function across both ectothermic and endothermic taxa with strong constraint on muscle-related metabolic roles. The expression levels of the *UCP3.L* and *UCP3.S* homoeologs were comparable, suggesting limited functional divergence at the transcriptional level. This conservation supports the hypothesis that UCP3 fulfills an ancestral muscle-related function, potentially associated with lipid substrate handling or protection against oxidative stress during sustained energetic demand (Della Guardia et al., 2024). Comparable expression levels of *UCP3.L* and *UCP3.S* further suggest limited divergence following whole-genome duplication. The organ-centric expression patterns observed here mirror the metabolic requirements of kidney and muscle tissues in amphibians exposed to fluctuating environmental conditions.

Across all *UCP* paralogs, homoeolog-specific differences in *UCP2* expression were evident, although the dominant homoeolog varied by gene and tissue. These patterns indicate asymmetric regulation of L and S subgenome copies and suggest that selective pressures following whole-genome duplication have acted at the level of transcriptional control rather than gene retention alone. Whether there is also some degree of asymmetry in the regulation of UCP1 and UCP3 homeolog expression cannot be fully resolved, given the limitations in the sample size of this study. The continued presence of all *UCP* homoeologs indicates that neither functional redundancy nor genome restructuring has led to substantial gene loss within this family, at least in *X. laevis*. Retention of both L and S homoeologs may reflect dosage sensitivity or complementary regulatory roles that constrain post-duplication gene loss. Such patterns are consistent with models of subfunctionalization, in which duplicated genes partition ancestral regulatory elements rather than acquiring entirely novel functions. The strong protein-level conservation observed between homoeologs further supports the idea that selective pressures act primarily on gene regulation rather than coding sequence divergence within the UCP family in amphibians.

### Implications for ancestral UCP function

Collectively, these findings support a model in which UCPs in ectothermic vertebrates primarily fulfill metabolic functions aligned with tissue-specific energetic demands. The high expression of *UCP1* and *UCP2* in the kidney and *UCP3* in muscle indicates that ancestral UCP functions were closely linked to mitochondrial regulation in organs with sustained metabolic activity. In contrast to the pioneering study on amphibian UCPs nearly 20 years ago (Trzcionka et al., 2008), we have now detected substantial levels of *UCP1* mRNA in kidneys, and new genome assemblies of *X. laevis* enabled us to resolve the UCP2-UCP3 genomic locus. While the previous study linked environmental stressors, such as cold exposure and fasting, to changes in *UCP2* or *UCP3* expression, our data indicate that baseline tissue expression already reflects physiological roles in energy metabolism. Importantly, by resolving homoeolog-specific expression, our study highlights the need to consider subgenome identity when interpreting gene function in polyploid organisms. Differential expression between L and S homoeologs may influence physiological outcomes without necessitating changes in protein sequence or biochemical activity.

By resolving homoeolog-specific genomic organization and expression in an allotetraploid vertebrate, this study highlights the importance of considering genome duplication when interpreting mitochondrial gene function. Differential regulation of L and S homoeologs may influence physiological outcomes without many changes in protein sequence, a principle likely applicable to other duplicated metabolic genes in polyploid organisms. Future studies integrating protein-level analyses and *in vivo* physiological measurements will be crucial for elucidating the precise mechanisms by which UCPs contribute to amphibian metabolic homeostasis.

In summary, our results establish a concise comparative framework for studies into the understanding of UCP biology outside the Mammalia lineage. *X. laevis* emerges as a powerful model for exploring the ancestral functions of mitochondrial carrier proteins, providing insight into how metabolic flexibility was later repurposed during the evolution of vertebrate thermoregulation.

## MATERIALS AND METHODS

### Animals

Adult female *X. laevis* frogs (1 year of age) were obtained from Nasco (Fort Atkinson, WI, USA) in 2020. Animals were maintained at 22°C under a 12 h light–dark cycle in recirculating freshwater systems and fed daily with frozen bloodworm cubes (*Chironomidae* larva; FBWC7, Brine Shrimp Direct). Animals were euthanized by prolonged anesthesia in ice-cold water followed by decapitation. Tissue dissection was performed on ice immediately after euthanasia. All procedures were conducted in accordance with institutional and national ethical guidelines and were approved by the Stockholm North Ethical Committee (permit number N69/15). Frozen tissues stored at −80°C from six deceased animals were taken for molecular analyses.

### Comparative genomics

Comparative genomic analyses were performed to assess the conserved synteny and homoeolog retention of *UCP1*, *UCP2*, and *UCP3* in *X. laevis*. Predicted *X. laevis* gene sequences and their neighboring loci were retrieved from NCBI and Ensembl databases (Xenopus_laevis_J-strain_v10.1, Ensembl annotation release 101, 14.05.2021, *International Xenopus Sequencing Consortium*). Orthologous loci from *H. sapiens* (GRCh38.p14, annotation release RS_2023_10, 03.02.2022, *Genome Reference Consortium*) and *D. rerio* (GRCz11, annotation release 106, 05.09.2017, *Genome Reference Consortium*) were used as references.

Nucleotide and protein sequence alignments were conducted using BLAST tools (https://blast.ncbi.nlm.nih.gov/Blast.cgi). Amino acid identity and query coverage values were used to support ortholog and homoeolog assignments. Gene order, transcriptional orientation, and neighboring gene conservation were manually curated to assess syntenic relationships. All analyses were conducted using annotated reference sequences and did not involve experimental genome manipulation. Various alignment metrics are shown in Tables S1 and S2, including accession numbers and gene annotation status.

### RNA isolation and cDNA synthesis

Total RNA was extracted from pulverized, frozen *X*. *laevis* tissues using QIAzol lysis reagent (79306, Qiagen) according to the manufacturer's instructions. RNA quantity and purity were assessed spectrophotometrically using NanoDrop One (ND-ONE-W; Thermo Fisher Scientific) according to the manufacturer’s instructions. cDNA was synthesized from 1 µg of total RNA using the QuantiTect Reverse Transcription Kit (205314, Qiagen), including genomic DNA elimination steps.

### Quantitative PCR and gene expression analysis

Quantitative real-time PCR (qPCR) was performed using gene-specific primers (see Table S3) designed to distinguish *UCP* paralogs and L and S homoeologs based on divergent 5′ and 3′ UTRs. Amplification reactions were carried out using SYBR Green Master Mix (S4438, Sigma) on a CFX384 Touch Real-Time PCR Detection System (1855484, Bio-Rad). Gene expression levels were calculated using the ΔCt method. The housekeeping gene *RPL8.L* was used for normalization based on expression stability across tissues. All qPCRs were performed in technical duplicates. Primer efficiencies were tested in technical triplicates using dilutions (0.01–50 ng/µl) of tissue sample mix from all frogs.

### Statistics

Data are presented as means±s.e.m. Gene expression was analyzed using ΔCt values (cycle threshold), which are depicted as 2^−ΔCt^. ΔCt was calculated as Ct target gene minus Ct reference gene. If there was no detection, the Ct was set to the highest cycle of 40, +1 (=41). Note that *UCP3.L* expression in the liver and spleen was below the detection threshold and was therefore considered as not detected in the figure but included in the statistical analysis. Outliers were identified and removed in GraphPad Prism (Version 11.0.0, GraphPad Software, 2025) before statistical analysis. *UCP1*, *UCP2* and *UCP3* gene expression data were tested for normality using the D'Agostino and Pearson test. To assess whether *UCP* expression differed between homoeologs, nonparametric multiple Mann–Whitney *U* tests were performed and corrected for multiple comparisons using the Holm–Sidak method.

## Supplementary Material



10.1242/biolopen.062691_sup1 Supplementary information
