# Supplementary material for: Retention and tissue-specific expression of uncoupling protein homoeologs in Xenopus laevis
Source: Biol Open. 2026 Jul 8;15(7):bio062691. doi: 10.1242/bio.062691 (PMC13382700; doi:10.1242/bio.062691)
Supplement: Supplementary information [file biolopen-15-062691-s1.pdf]

Table S1. Nucleotide and protein alignments of genes in the *Xenopus laevis*, *Homo sapiens*, and *Danio rerio UCP1* loci.

	Query/Subject Information				Nucleotide BLAST						Protein BLAST					
	Gene / Predicted Gene (Query on top)		Species	NCBI RefSeq (Provisional/ Validated)	Max Score	Total Score	Query Cover	E Value	Identity	Acc. Length	Max Score	Total Score	Query Cover	E Value	Identity	Acc. Length
TBC1D9	*LOC108699540*<br>*LOC108706674*	***TBC1D9.L***<br>***TBC1D9.S***	***X. laevis***<br>***X. laevis***	XM_018231640.2 (**P**)<br>XM_018243287.2 (**P**)	5768	5768	**85%**	0,0	**93,50%**	4492	2477	2477	**100%**	0,0	**96,35%**	1233
TBC1D9	***TBC1D9***<br>*LOC108699540*	 ***TBC1D9.L***	***D. rerio***<br>***X. laevis***	XM_686942.9 (**P**)<br>XM_018231640.2 (**P**)	1915	2078	**67%**	0,0	**74,85%**	4518	1914	1914	**98%**	0,0	**77,99%**	1227
TBC1D9	***TBC1D9***<br>*LOC108706674*	 ***TBC1D9.S***	***D. rerio***<br>***X. laevis***	XM_686942.9 (**P**)<br>XM_018243287.2 (**P**)	1885	2100	**70%**	0,0	**74,74%**	4492	1895	1895	**98%**	0,0	**76,74%**	1234
TBC1D9	***TBC1D9***<br>*LOC108699540*	 ***TBC1D9.L***	***H. sapiens***<br>***X. laevis***	NM_015130.3 (**V**)<br>XM_018231640.2 (**P**)	2439	2854	**62%**	0,0	**78,75%**	4518	2071	2071	**98%**	0,0	**83,67%**	1227
TBC1D9	***TBC1D9***<br>*LOC108706674*	 ***TBC1D9.S***	***H. sapiens***<br>***X. laevis***	NM_015130.3 (**V**)<br>XM_018243287.2 (**P**)	2376	2376	**76%**	0,0	**78,23%**	5554	2038	2038	**98%**	0,0	**82,46%**	1233
UCP1	***UCP1.L***<br>***UCP1.S***		***X. laevis***<br>***X. laevis***	NM_001097741.1 (**P**)<br>NM_001095178.1 (**P**)	1565	1850	**88%**	0.0	**92,34%**	1732	621	621	**100%**	0,0	**95,48%**	310
UCP1	***UCP1***<br>***UCP1.L***		***D. rerio***<br>***X. laevis***	NM_199523.2 (**P**)<br>NM_001097741.1 (**P**)	453	453	**36%**	3e-130	**71,38%**	1776	519	519	**100%**	0,0	**78,71%**	310
UCP1	***UCP1***<br>***UCP1.S***		***D. rerio***<br>***X. laevis***	NM_199523.2 (**P**)<br>NM_001095178.1 (**P**)	435	435	**36%**	3e-124	**70,82%**	1732	521	521	**100%**	0,0	**78,06%**	310
UCP1	***UCP1***<br>***UCP1.L***		***H. sapiens***<br>***X. laevis***	NM_021833.5 (**V**)<br>NM_001097741.1 (**P**)	304	304	**53%**	7e-86	**67,85%**	1776	402	402	**100%**	1e-146	**63,14%**	310
UCP1	***UCP1***<br>***UCP1.S***		***H. sapiens***<br>***X. laevis***	NM_021833.5 (**V**)<br>NM_001095178.1 (**P**)	270	270	**53%**	1e-75	**67,01%**	1732	408	408	**100%**	8e-149	**63,02%**	310
ELMOD2	***ELMOD2.L***<br>*LOC108704374*	 ***ELMOD2.S***	***X. laevis***<br>***X. laevis***	NC_054371.1 (**P**)<br>108704374 (**P**)	864	2454	**19%**	0,0	**98,20%**	5107	Only genomic sequence for *LOC108706674* / *ELMOD2.S* available					
ELMOD2	***ELMOD2***<br>***ELMOD2.L***		***D. rerio***<br>***X. laevis***	NM_001204132.1 (**V**)<br>NC_054371.1 (**P**)	261	291	**10%**	4e-72	**70,88%**	2626	397	397	**97%**	5e-145	**63,01%**	293
ELMOD2	***ELMOD2***<br>***ELMOD2.L***		***H. sapiens***<br>***X. laevis***	NM_153702.4 (**V**)<br>NC_054371.1 (**P**)	397	397	**31%**	6e-113	**71,43%**	4399	410	410	**98%**	4e-150	**67,47%**	293
ELMOD2	*LOC108699553*<br>*LOC108704374*	***ncRNA***<br>***ELMOD2.S***	***X. laevis***<br>***X. laevis***	108699553 (**P**)<br>108704374 (**P**)	No significant similarity found						Only genomic sequence for *LOC108706674* / *ELMOD2.S* available					
MGAT4D	***MGAT4D.L***<br>***MGAT4D.S***		***X. laevis***<br>***X. laevis***	XM_018231673.2 (**P**)<br>NM_001095836.1 (**P**)	2759	2759	**82%**	0,0	**90,73%**	2074	1023	1023	**100%**	0,0	**92,61%**	537
MGAT4D	*LOC108190370*<br>***MGAT4D.L***	uncharacterized 	***D. rerio***<br>***X. laevis***	NC_007112.7<br>NC_054371.1 (**P**)	35,6	35,6	**0%**	0,005	**91,67%**	64562	No significant similarity found					
MGAT4D	*LOC108190370*<br>***MGAT4D.S***	uncharacterized 	***D. rerio***<br>***X. laevis***	NC_007112.7<br>NC_054372.1 (**P**)	No significant similarity found						No significant similarity found					
MGAT4D	***MGAT4D***<br>***MGAT4D.L***		***H. sapiens***<br>***X. laevis***	NC_000004.12 (**V**)<br>NC_054371.1 (**P**)	61,7	143	**6%**	3e-12	**89,58%**	2370	281	281	**98%**	1e-94	**37,90%**	528
MGAT4D	***MGAT4D***<br>***MGAT4D.S***		***H. sapiens***<br>***X. laevis***	NC_000004.12 (**V**)<br>NC_054372.1 (**P**)	75,2	75,2	**10%**	1e-16	**67,77%**	2074	283	283	**99%**	2e-95	**38,90%**	529

Biology Open • Supplementary information

Table S2. Nucleotide and protein alignments of genes in the *Xenopus laevis*, *Homo sapiens*, and *Danio rerio* UCP2 / UCP3 loci.

	Query/Subject Information				Nucleotide BLAST						Protein BLAST					
	Gene / Predicted Gene (Query on top)		Species	NCBI RefSeq (Provisional/ Validated)	Max Score	Total Score	Query Cover	E Value	Identity	Acc. Length	Max Score	Total Score	Query Cover	E Value	Identity	Acc. Length
**C2CD3**	*LOC121400083*	***ncRNA***	***X. laevis***	XR_005965543.1 (P)	No significant similarity found						No significant similarity found					
	C2CD3.S		***X. laevis***	XM_018250436.2 (P)												
	LOC110440075	***ncRNA***	***D. rerio***	XR_002459626.1 (P)	No significant similarity found						No significant similarity found					
	LOC121400083	***ncRNA***	***X. laevis***	XR_005965543.1 (P)												
	LOC110440075	***ncRNA***	***D. rerio***	XR_002459626.1 (P)	No significant similarity found						No significant similarity found					
	C2CD3.S		***X. laevis***	XM_018250436.2 (P)												
	C2CD3		***H. sapiens***	NM_001286577.2 (V)	No significant similarity found						No significant similarity found					
	LOC121400083	***ncRNA***	***X. laevis***	XR_005965543.1 (P)												
	C2CD3		***H. sapiens***	NM_001286577.2 (V)	266	979	**38%**	4e-73	**65,00%**	7939	1847	1847	**98%**	0,0	**45,27%**	2354
	C2CD3.S		***X. laevis***	XM_018250436.2 (P)												
**UCP3**	*LOC108707628*	***UCP3.L***	***X. laevis***	XM_041582790.1 (P)	2076	2076	**99%**	0,0	**80,43%**	2239	585	585	**99%**	0,0	**91,83%**	310
	LOC108709041	***UCP3.S***	***X. laevis***	XM_018248668.2 (P)												
	UCP3		***D. rerio***	NM_200353.2 (V)	402	402	**41%**	4e-115	**70,23%**	1666	478	478	**99%**	2e-176	**72,96%**	310
	LOC108707628	***UCP3.L***	***X. laevis***	XM_041582790.1 (P)												
	UCP3		***D. rerio***	NM_200353.2 (V)	383	383	**39%**	1e-109	**69,96%**	1666	490	490	**100%**	0,0	**74,19%**	310
	LOC108709041	***UCP3.S***	***X. laevis***	XM_018248668.2 (P)												
	UCP3		***H. sapiens***	NM_003356.4 (V)	374	374	**41%**	8e-107	**69,57%**	2277	448	565	**97%**	1e-164	**70,86%**	313
	LOC108707628	***UCP3.L***	***X. laevis***	XM_041582790.1 (P)												
	UCP3		***H. sapiens***	NM_003356.4 (V)	355	355	**40%**	8e-101	**69,41%**	2277	451	451	**100%**	8e-166	**69,33%**	313
	LOC108709041	***UCP3.S***	***X. laevis***	XM_018248668.2 (P)												
**UCP2**	***UCP2.L***		***X. laevis***	NM_001086754.1 (P)	1910	2124	**98%**	0,0	**90,05%**	2010	621	621	**100%**	0,0	**96,43%**	308
	UCP2.S		***X. laevis***	NM_001091378.1 (P)												
	UCP2		***D. rerio***	NM_131176.1 (P)	604	604	**44%**	6e-176	**75,05%**	1501	535	535	**100%**	0,0	**81,67%**	311
	UCP2.L		***X. laevis***	NM_001086754.1 (P)												
	UCP2		***D. rerio***	NM_131176.1 (P)	585	614	**45%**	2e-170	**74,50%**	1501	537	537	**100%**	0,0	**81,67%**	311
	UCP2.S		***X. laevis***	NM_001091378.1 (P)												
	UCP2		***H. sapiens***	NM_001381943.1 (V)	603	603	**46%**	7e-176	**74,76%**	1903	534	534	**100%**	0,0	**83,23%**	310
	UCP2.L		***X. laevis***	NM_001086754.1 (P)												
	UCP2		***H. sapiens***	NM_001381943.1 (V)	572	572	**46%**	1e-166	**73,88%**	1902	531	531	**100%**	0,0	**82,26%**	310
	UCP2.S		***X. laevis***	NM_001091378.1 (P)												
**UCP2/3**	*LOC108707628*	***UCP3.L***	***X. laevis***	XM_041582790.1 (P)	No significant similarity found						480	480	**99%**	3e-177	**75,16%**	308
	UCP2.L		***X. laevis***	NM_001086754.1 (P)												
	LOC108709041	***UCP3.S***	***X. laevis***	XM_018248668.2 (P)	No significant similarity found						485	485	**100%**	3e-179	**74,84%**	308
	UCP2.S		***X. laevis***	NM_001091378.1 (P)												
**DNAJB13**	*LOC108707627*	***ZNF250.L***	***X. laevis***	XM_041582784.1 (P)	111	111	**3%**	9e-28	**85,87%**	1070	No significant similarity found					
	DNAJB13.S		***X. laevis***	NM_001096424.1 (P)												
	DNAJB13		***D. rerio***	NM_001017606.1 (V)	229	229	**73%**	2e-63	**67,54%**	1107	444	444	**99%**	6e-163	**68,79%**	323
	DNAJB13.S		***X. laevis***	NM_001096424.1 (P)												
	DNAJB13		***H. sapiens***	NM_001377263.1 (V)	285	285	**70%**	3e-80	**68,47%**	1293	388	388	**81%**	1e-141	**70,54%**	259
	DNAJB13.S		***X. laevis***	NM_001096424.1 (P)												

Table S3. Primer Sequences and Efficiencies.

Primer Name	Forward Sequence	Reverse Sequence	Primer Efficiency (%)
RPL8.L	**TGCCACAGTTATCTCCCACA**	**ATACGACCACCTCCAGCAAC**	**100.69**
UCP1.L	**TCCTAAACGTCCACTGCATCC**	**GCGCAACTGACCATTCATTG**	**99.31**
UCP1.S	**ATTCCAGCAATCCCCAACCTGT**	**TGCCAGCATCCAGTTACTGTTG**	**115.94**
UCP2.L	**CACCTAGACTGGGGGTTGCAAT**	**ACCAGCACTGCAGAGATTACGA**	**127.41**
UCP2.S	**CGGAGCTGGTCACCTATGATC**	**AGGCGGAGGTGAAATGACAG**	**92.70**
UCP3.L	**TTTACCTTCCCCCTTGACACTG**	**TCAATGCATGATGGAATAAAA**	**103.67**
UCP3.S	**TTTACCTTTCCCCTCGACACTG**	**CCCTTGTACCGCAGAACTTTG**	**143.86**
