# Supplementary material for: Retention and tissue-specific expression of uncoupling protein homoeologs in Xenopus laevis
Source: Biol Open. 2026 Jul 8;15(7):bio062691. doi: 10.1242/bio.062691 (PMC13382700; doi:10.1242/bio.062691)
Supplement: Supplementary file 1 [file biolopen-15-062691_review_history.pdf]

Retention and tissue-specific expression of uncoupling protein homoeologs in Xenopus laevis

Erik Rollwitz and Martin Jastroch
10.1242/bio.062691

Editor: Nicholas Wu

Review timeline

Original submission:	11 May 2026
Editorial decision:	22 May 2026
First revision received:	5 June 2026
Accepted:	8 June 2026

Original submission

First decision letter

MS ID#: bio.062691

MS Title: Retention and tissue-specific expression of uncoupling protein homoeologs in Xenopus laevis

Authors: Erik Rollwitz; Martin Jastroch

I have now reached a decision on the above manuscript.

The reviewer reports are shown at the bottom of this email.

As you will see, the reviewers gave favourable reports, but raised some critical points that will require amendments to your manuscript. I hope that you will be able to carry these out, because we would like to be able to accept your paper. Both reviewers found the paper to be of high quality and well written. I agree with reviewer 2 that the sample size is small for statistical analysis. Please justify the sample size used and how it compares to similar studies (provide some citations from other authors) in the field.

At this stage, we also ask you to ensure your manuscript complies with our formatting guidelines - please see our manuscript preparation guidelines for details. Provided you are able to fully address the referees' comments, we are positive about publication of your paper (we accept over 95% of revision submissions) and therefore hope you won't mind any extra work involved in reformatting your manuscript at this point.

Please upload both a 'clean' version of your Word file, along with a highlighted version clearly showing where you have made changes in the revised manuscript. Please avoid using 'Track changes' in Word files as these are lost in PDF conversion.

I should be grateful if you would also provide a point-by-point response detailing how you have dealt with the points raised by the reviewers in the 'Response to Reviewers' box. Please attend to all of the reviewers' comments. If you do not agree with any of their criticisms or suggestions please explain clearly why this is so.

Reviewer 1

© 2026. Published by The Company of Biologists under the terms of the Creative Commons Attribution License (https://creativecommons.org/licenses/by/4.0/).

Comments for the author

Mitochondrial uncoupling proteins (UCPs) are important regulators of cellular energy metabolism and mitochondrial function. In this study, the authors investigated the genomic organization, conserved synteny, and tissue-specific expression of the three UCP paralogs and their homologs in Xenopus laevis. Their analyses revealed distinct expression patterns, with UCP1 and UCP2 predominantly expressed in the kidney and UCP3 mainly enriched in muscle, highlighting their association with energetically demanding tissues.

A major contribution of this work is the demonstration that UCP genes are evolutionarily conserved in amphibians, supporting the idea that their ancestral function was primarily linked to metabolic regulation rather than thermogenesis. By establishing X. laevis as a comparative model for UCP biology outside mammals, the study provides an important framework for investigating the evolutionary origins of mitochondrial energy regulation and the later emergence of vertebrate thermoregulatory mechanisms. Although the study is primarily descriptive, it is very well written and logically organized. The figures are clear, well designed, and easy to follow, allowing the reader to readily understand the experimental approaches and conclusions. Importantly, the data convincingly support the authors' interpretations. The evolutionary significance of studying UCPs in X. laevis is also well explained throughout the manuscript, emphasizing the relevance of this amphibian model for understanding the evolution of mitochondrial metabolism and vertebrate physiology.

Reviewer 2

Comments for the author

This study investigated the retention and differential expression of UCP paralogs/homoeologs in the allotetraploid X. laevis. It advances previous work by resolving the UCP2/UCP3 genomic organization and quantifying homoeolog-specific expression patterns using updated Xenopus laevis genome resources. The paper is written extremely well, and although the sample size is rather low, the findings contribute to our broader understanding of the role of UCPs in vertebrates. I only have minor comments I would like the authors to address:

1. Statistics: did the authors check for normal distribution before using the parametric test?

2. The sample size is reported as 6 in the methods, but "n=2-6" in Fig 3. Why do some tissues have a lower sample size, and how could a parametric ANOVA be run under these conditions?

3. The figure legend for Fig 3 reports that the "Data are shown as individual values" - I accept my eyes are failing, but the size of the individual data points is incredibly small. Suggest increasing for clarity.

4. Line 14, page 5: Suggest also divergence relative to human UCP1 alongside the zebrafish comparison

5. Suggest including sample size as a limitation somewhere

Reviewer's Responses to Questions

Experimental quality
Are experiments performed using appropriate methods that will answer the question (or test the hypothesis or support the observations) posed by the authors? Is the right tool used for the job?
If 'No', please indicate reasons in Comments for Author box below.
Reviewer #1:

- Yes

Reviewer #2:

© 2026. Published by The Company of Biologists under the terms of the Creative Commons Attribution License (https://creativecommons.org/licenses/by/4.0/).

- Yes

Were the data analyzed using appropriate statistical tests?
If 'No', please indicate reasons in Comments for Author box below.
Reviewer #1:

- Yes

Reviewer #2:

- No

Reproducibility
Were experiments performed using adequate number of biological replicates?
If 'No', please indicate reasons in Comments for Author box below.
Reviewer #1:

- Yes

Reviewer #2:

- No

Is there sufficient raw data to assess the rigor of the analysis?
If 'No', please indicate reasons in Comments for Author box below.
Reviewer #1:

- Yes

Reviewer #2:

- Yes

Does the methods section provide sufficient detail to permit reproducibility?
If 'No', please indicate reasons in Comments for Author box below.
Reviewer #1:

- Yes

Reviewer #2:

- Yes

Completeness
Are the manuscript's conclusions supported by the data?
If 'No', please indicate reasons in Comments for Author box below.
Reviewer #1:

- Yes

Reviewer #2:

- Yes

Scholarship
Do the authors cite and discuss the merits of data that would argue for and against their conclusion?
If 'No', please indicate reasons in Comments for Author box below.
Reviewer #1:

- Yes

Reviewer #2:

- Yes

© 2026. Published by The Company of Biologists under the terms of the Creative Commons Attribution License (https://creativecommons.org/licenses/by/4.0/).

Does the manuscript title & abstract accurately reflect the contents of the manuscript, without hyperbole?
If 'No', please indicate reasons in Comments for Author box below.
Reviewer #1:
- Yes

Reviewer #2:
- Yes

First revision

Author response to reviewers' comments

We thank the handling editor and both reviewers for their time, and for providing positive and constructive feedback on our paper. We have addressed all comments and implemented the suggested changes to the best of our ability, believing that this improved the overall quality of the manuscript. We listed our responses point-to-point below.

Reviewer 1: Mitochondrial uncoupling proteins (UCPs) are important regulators of cellular energy metabolism and mitochondrial function. In this study, the authors investigated the genomic organization, conserved synteny, and tissue-specific expression of the three UCP paralogs and their homologs in Xenopus laevis. Their analyses revealed distinct expression patterns, with UCP1 and UCP2 predominantly expressed in the kidney and UCP3 mainly enriched in muscle, highlighting their association with energetically demanding tissues.

A major contribution of this work is the demonstration that UCP genes are evolutionarily conserved in amphibians, supporting the idea that their ancestral function was primarily linked to metabolic regulation rather than thermogenesis. By establishing X. laevis as a comparative model for UCP biology outside mammals, the study provides an important framework for investigating the evolutionary origins of mitochondrial energy regulation and the later emergence of vertebrate thermoregulatory mechanisms. Although the study is primarily descriptive, it is very well written and logically organized. The figures are clear, well designed, and easy to follow, allowing the reader to readily understand the experimental approaches and conclusions. Importantly, the data convincingly support the authors' interpretations. The evolutionary significance of studying UCPs in X. laevis is also well explained throughout the manuscript, emphasizing the relevance of this amphibian model for understanding the evolution of mitochondrial metabolism and vertebrate physiology.

RESPONSE: We thank the reviewer for the positive feedback and highlighting the main findings and the novelty of our study.

Reviewer 2: This study investigated the retention and differential expression of UCP paralogs/homoeologs in the allotetraploid X. laevis. It advances previous work by resolving the UCP2/UCP3 genomic organization and quantifying homoeolog-specific expression patterns using updated Xenopus laevis genome resources. The paper is written extremely well, and although the sample size is rather low, the findings contribute to our broader understanding of the role of UCPs in vertebrates. I only have minor comments I would like the authors to address:

1. Statistics: did the authors check for normal distribution before using the parametric test?
2. The sample size is reported as 6 in the methods, but "n=2-6" in Fig 3. Why do some tissues have a lower sample size, and how could a parametric ANOVA be run under these conditions?

RESPONSE: We thank the reviewer for identifying these important short-comings, which prompted us to re-analyze some of our data and specify the procedures. We corrected our description of

© 2026. Published by The Company of Biologists under the terms of the Creative Commons Attribution License (https://creativecommons.org/licenses/by/4.0/).

sample size, statistics and report this transparently in the revision.

a. As stated in the MM section, frozen tissues of 6 individual frogs were used. The isolated RNA of two kidney and one pancreas samples did not reach sufficient quality, reducing the minimum n-value to 4.
b. All other tissues yielded in RNA and cDNA, but UCP expression levels in some samples were below detection levels, despite good detection of the housekeeping gene. We previously omitted samples with no detection for statistical evaluation but learnt that it is standard to estimate the expression and include the values in the statistics, as now described in the MM Statistics section. The data were checked for normal distribution and required a non-parametric test.
c. Furthermore, we performed outlier tests on the PCR data, where in some case one sample of a group had to be omitted. The minimum n-value remained 4.

We made the following amendments:

1. We report the final n-values in the legend of Fig. 3: Mann-Whitney tests were performed and corrected for multiple comparisons using the Holm-Sidak method; *n* = 4-6.

2. 3.5. Statistics
Data are presented as means ± SEM. Gene expression was analyzed using ΔCt (cycle threshold) values and are depicted as 2^- ΔCt. ΔCt was calculated as Ct target gene minus Ct reference gene. If there was no detection, the Ct was set to the highest cycle of 40, + 1 (=41). Note that *UCP3.L* expression in liver and spleen was below the detection threshold and was therefore considered as not detected in the figure but included in the statistical analysis. Outliers were identified and removed in GraphPad Prism (Version 11.0.0, GraphPad Software, 2025) before statistical analysis. UCP1, UCP2 and UCP3 gene expression data were tested for normality using the D'Agostino and Pearson test. To assess whether UCP expression differed between homoeologs, non-parametric multiple Mann-Whitney tests were performed and corrected for multiple comparisons using the Holm-Sidak method.

3. The figure legend for Fig. 3 reports that the "Data are shown as individual values" - I accept my eyes are failing, but the size of the individual data points is incredibly small. Suggest increasing for clarity.

RESPONSE: We apologize and increased the data symbols for clarity.

4. Line 14, page 5: Suggest also divergence relative to human UCP1 alongside the zebrafish comparison

RESPONSE: Thanks for pointing this out. We included the percentage deviation from human UCP1.

5. Suggest including sample size as a limitation somewhere

RESPONSE: We added this in the expression section, linked to conclusions on homeolog differences: Whether there is also some degree of asymmetry in the regulation of UCP1 and UCP3 homeolog expression cannot be fully resolved, given the limitations in the sample size of this study.

Second decision letter

MS ID#: bio.062691R1

MS Title: Retention and tissue-specific expression of uncoupling protein homoeologs in Xenopus laevis

Authors: Erik Rollwitz; Martin Jastroch

© 2026. Published by The Company of Biologists under the terms of the Creative Commons Attribution License (https://creativecommons.org/licenses/by/4.0/).

I am happy to tell you that your manuscript has been accepted for publication in Biology Open, pending our standard publication integrity checks. It was accepted on 8th June 2026.

© 2026. Published by The Company of Biologists under the terms of the Creative Commons Attribution License (https://creativecommons.org/licenses/by/4.0/).
